# Evaluation of relationship between bicuspid aortic valve phenotype with valve dysfunction and associated aortopathy

**DOI:** 10.15171/jcvtr.2018.41

**Published:** 2018-12-13

**Authors:** Mehrnoush Toufan Tabrizi, Roghaieh Rahimi Asl, Soheyla Nazarnia, Leili Pourafkari

**Affiliations:** Cardiovascular Research Center, Tabriz University of Medical Sciences, Tabriz, Iran

**Keywords:** Bicuspid Aortic Valve, Valvular Dysfunction, Aortopathy

## Abstract

***Introduction:*** Morphology of bicuspid aortic valve (BAV) may have implication in the associated pathologies including aortic stenosis (AS), aortic insufficiency (AI) and aortic dilation. The aim of this study is to investigate the frequency and patterns of valvular dysfunction and aortopathy associated with different phenotypes of BAV in a referral center in northwest of Iran.

***Methods:*** In this prospective study patients who presented to our echocardiography lab between January 2014 and December 2015 and were diagnosed with BAV were assessed. Frequency of various BAV phenotypes and their association with valvular dysfunction and aortopathy was evaluated. A P value less than 0.05 was considered statistically significant.

***Results: *** The average age of the study patients was 40±16 years, with predominance of male sex (72%). Patients with anteroposteriorly located BAV (BAV-AP) phenotype constituted majority of our cases with prevalence of 62.7%, while 37.3% of cases had right-left (BAV-RL) located valves. BAV-RL patients when compared to BAV-AP patients had higher frequencies of dilated aortic arch (25% vs. 4.3%, *P* < 0.001), AS (56.3% vs. 31.4%, *P* < 0.001), mass or vegetation on aortic valve (14.3 vs. 6.4%, *P* = 0.023) and lower frequencies of dilated aortic root (42.9% vs. 57.4%, *P* = 0.01), aortic insufficiency (68.8% vs. 79.8%, *P* = 0.034) and co-arctation of aorta (3.6% vs. 11.7%, *P* = 0.01).

***Conclusion: *** There seems to be a relationship between various BAV phenotypes, and frequency and pattern of aortic valve dysfunction and aortopathy. These findings suggest that examining leaflet morphology in BAV might help in risk stratification of these patients.

## Introduction

Bicuspid aortic valve (BAV) is the most common congenital heart disease and occurs in 2.8% of general population^1,2^ BAV appears to be inherited in an autosomal dominant fashion with incomplete penetrance. It has been postulated that the defective genes encoding the protein matrix structure, could be responsible for developmental impairment of heart, leading to valvular abnormalities.^3-5^ Common embryonic origins of the aortic valve and ascending aorta, may explain the existing association of BAV with different types of aortopathies. Pathophysiology of BAV at genetic level is also known to be related with other congenital heart diseases such as patent ductus arteriosus (PDA), coarctation of aorta and aortic aneurysm. Patients with BAV have a high incidence of aortic valve stenosis (AS), aortic valve insufficiency (AI), aortic aneurysm, aortic dissection and infected endocarditis.^3,6-8^

Studies in literature have suggested that 33% of patients with BAV will suffer serious and life-threatening complications in their lifetime. Therefore, early detection and prevention of complications of BAV are of paramount importance.^6^ It seems that, not all the patients with BAV manifest valvular dysfunction. Additionally, valvular dysfunction in patients with BAV appears to follow a wide clinical spectrum. Some patients with BAV present with AS, while others demonstrate single-cusp aortic valve prolapse leading to valvular regurgitation without any stenosis. Concomitant valvular stenosis and insufficiency is another mode of presentation.^9,10^ BAV is classified as general types of anteroposteriorly located BAV (BAV-AP) and right-left (BAV-RL) located valves on the basis of fusion pattern of valve cusps. Phenotype BAV-AP results from fusion of right and left cusps and constitutes majority of the cases. Fusion of either right or left cusp with non-coronary cusp results in BAV-RL.^11^ Several studies have been conducted to study the association of different phenotypes of BAV with valvular dysfunctions (AI, AS) and aortopathies, results of which appear to be contradictory.^10,12-17^ Additionally, in children and young subjects, BAV-RL type was found to be associated with a higher risk of valvular dysfunction and earlier presentation of AS or AI.^9,10^ Thus far such association has not been established in adult patients.^12,18^

Present study is the first in our geographical region to investigate BAV phenotypes and its associations. Nevertheless, there remains, many other aspects of this pathology to be investigated. The aim of this study is to evaluate the BAV phenotypes and their relationship with patterns of valvular dysfunction and associated aortopathies using echocardiography.

## Materials and Methods

Each examination was conducted, after informed consent was obtained following a full explanation of the purpose of our study and its methodology. In this cross-sectional study, patients who presented to our echocardiography laboratory were evaluated between January 2014 and December 2015 and those with BAV were enrolled. BAV patients, who had a history of valve surgery or aortic root repair were excluded from the study. Enrolled patients underwent TTE examination and also Transesophageal echocardiography (TEE), if deemed necessary at the discretion of the cardiologist. All examinations were performed using Vivid (Vivid7, GE, Norway) or Medison7 (Echo7, Samsung, SouthKorea) (2.5-3.5 MHz) systems. All examinations were performed and interpreted under the supervision of two experienced cardiologists with mutual consensus.

Studies were done with patients placed on left lateral decubitus position and para-sternal and apical views were obtained for our assessment.

**Figure 1:**
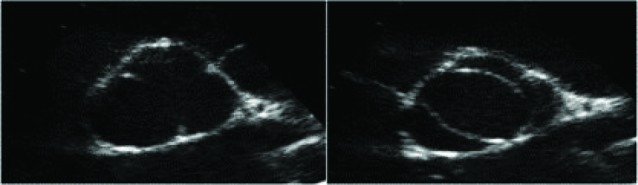
Bicuspid aortic valve with anteroposterior orientation.

**Figure 2:**
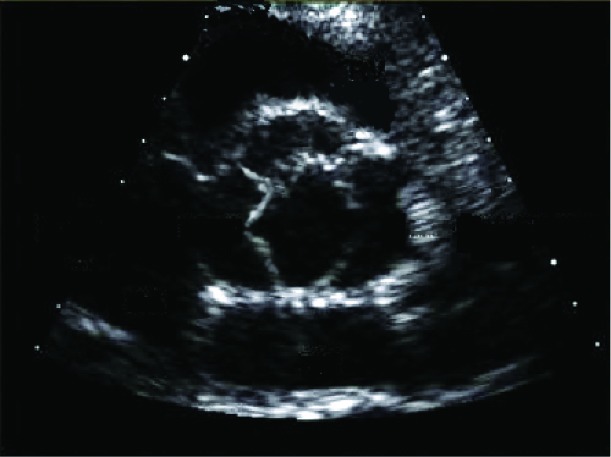
Bicuspid aortic valve with mediolateral orientation.

A comprehensive echocardiographic examination was performed with evaluation and measurements of atria and ventricles, aortic root, ascending aorta and aortic arch. Detailed evaluation of aortic valve morphology and pathology was done using two-dimensional echocardiography, color flow Doppler and spectral Doppler (Figure 1 and Figure 2). Presence of AS was established with valve gradients measured at apical 5 chamber view and presence of AI was diagnosed by color flow Doppler and descending aortic pulse wave Doppler for holo-diastolic reversal. The BAV phenotypes were identified, and aortic root and ascending aorta were examined for establishment of classification status. Subsequently, the incidence of aortic valve dysfunction in all BAV phenotypes were determined. The information obtained was stored in digital form to be used for offline analysis and data collection.

Variables for analysis included age, gender, echocardiographic measurements of both atria and ventricles, grades of left ventricular diastolic function, aortic valve phenotype, pattern and severity of valvular dysfunction and associated aortopathy. Phenotypes of BAV and aortopathy classifications used in this study are given in Table 1.^11^

**Table 1:** Types of bicuspid aortic valves and aortopathy

**Bicuspid aortic valve types**	Type 1: Antero-posterior (AP) caused by right coronary cusp (RCC) and left coronary cusp (LCC) fusion. Type 2: True AP, with no fusion, and no raphe, both cusps are equal and commensurate. Type 3: Mediolatral type with NCC and RCC fusion. Type 4: Mediolatral type with NCC and LCC fusion. Type 5: Mediolatral type with no fusion or raphe.
**Aortopathy types**	Type 0: Normal. Type 1: Aortic root dilatation. Type 2: Ascending aorta dilatation. Type 3: Ascending aorta dilatation extending to arch of aorta.

### Statistical Analysis

Continuous variables such as age are described as mean + SD and frequency variables are expressed as n (%). For comparison of frequency variables, chi-Square was used; and for comparison of continuous variables, one-way ANOVA and hoc-test was used. All statistical analyses were performed with SPSS version 22 statistical software. *P* value for significance was considered to be less than 0.05.

## Results

In this study, 300 adult patients with BAV consisted of 216 males (72%) and 84 females (28.5%) with mean age of 40 ± 16 years (range 14-76 years) were evaluated. Frequency of valvular phenotypes in study subjects with a diagnosis of BAV were as follows: Type 1 =163 patients (54.3%), type 2 =25 patients (8.3%), type 3 =74 patients (24.7%), type 4 = 3 patient (1%) and type 5=35 patient (11.7%). Patients with phenotype 1 and 3 constituted majority of the study participants. Due to low frequency of type 4 BAV (3 patients, 1%), these patients were not included in statistical analyses. All three patients diagnosed with type 4, were females, had normal diastolic function, and no AS. The valvular dysfunction in this group of patients was found to be mild to severe AI. Also, they all had type 1 aortopathy.

In this study, 121 (74.2%) of phenotype type 1 patients, 16 (64%) of phenotype type 2, 57 (77%) of phenotype type 3 and 22 (62.9%) of phenotype type 5 patients were males. In all the four phenotypes, there was a preponderance of male subjects, but there was no significant difference in this respect, when the four groups were compared (*P*=0.31). The most common aortopathy in all of the groups was found to be type 2 aortopathy followed by type 3. In patients with phenotype 2 BAV, the only aortopathy observed was type 2.

Table 2 demonstrates the echocardiographic findings of the BAV phenotypes in our study. Assessment of right ventricular end diastolic diameter (RVEDD) and right atrial (RA) diameter revealed statistically significant (*P* < 0.05) differences between phenotype 3 and all other analyzed phenotypes (1, 2 and 5). Number of patients with dilated aortic annulus in phenotypes 1 and 3 was comparable but higher than other phenotypes. Compared to other phenotypes, phenotype 5 had lower number of patients with dilated aortic root (*P*=0.002) and sinotubular junction (STJ) (*P*=0.05). There were no significant differences between any of the phenotypes, when dilatation of ascending aorta was assessed. Comparing the size of aortic arch between phenotypes, there were significant differences, between phenotype 3 and phenotype 1 (*P*<0.001), and also between phenotype 3 and phenotype 2 (*P*=0.013). It was noted that, the largest mean diameter of aortic arch was encountered in phenotypes 3 and 5 (*P* <0.001).

**Table 2:** Echocardiography findings according to the type of BAV phenotype

	**Phenotype1**	**Phenotype2**	**Phenotype3**	**Phenotype5**	***P*** ** value**
LVEDD	5.38± 1.06	5.43 ± 1.07	5.24 ± 1.03	5.18 ± 0.98	0.621
Abnormal LV size (%)	70 (43%)	15 (60%)	28 (38%)	13 (37%)	0.231
RVEDD	3.22 ± 0.31	3.18 ± 0.24	3.33 ± 0.37	3.20 ± 0.15	0.032
Abnormal RV size (%)	39 (24%)	4 (16%)	29 (39%)	4(11%)	0.006
LA diameter	3.47 ± 0.64	3.58 ± 0.66	3.54 ± 0.44	3.56 ± 0.44	0.683
Abnormal LA size (%)	29 (2%)	6 (24%)	18 (24%)	10 (29%)	0.423
RA diameter	3.18 ± 0.27	3.10 ± 0.25	3.28 ± 0.32	3.14 ± 0.20	0.012
Abnormal RA size (%)	2 (1%)	Zero	6 (7%)	Zero	0.433
IVSD	10.76 ± 2.52	9.92 ± 1.52	11.06 ± 2.41	10.80 ± 1.82	0.220
Annulus of Aorta	2.46 ± 0.37	2.30 ± 0.30	2.48 ± 0.36	2.42 ± 0.26	0.155
Dilated annulus (%)	20 (12%)	1 (4%)	12 (16%)	3 (9%)	0.373
Aortic root	3.57 ± 0.49	3.42 ± 0.53	3.53 ± 0.42	3.41 ± 0.49	0.192
Dilated aortic root (%)	90 (55 %)	11 (44%)	40 (54%)	7 (20%)	0.002
STJ size	3.19 ± 0.48	3.09 ± 0.42	3.28 ± 0.55	3.06 ± 0.59	0.100
Dilated STJ (%)	61 (37%)	9 (36%)	31 (42%)	3 (9%)	0.005
Ascending Aorta	3.99 ± 0.76	3.70 ± 0.56	4.07 ± 0.73	3.83 ± 0.83	0.117
Dilated AAO (%)	118 (72%)	15 (60%)	54(72%)	25 (71%)	0.621

AAO: ascending aorta, IVSD: inter-ventricular septum diameter, LA: left atrium, LV: left ventricle, LVEDD: left ventricular end diastolic diameter, RA: right atrium, RV: right ventricle, RVEDD: right ventricular diameter, STJ: sinotubular junction

Table 3 demonstrates valvular findings in different phenotypes of BAV. There was a significant difference in the incidence of AS (*P*<0.001) and AI (*P* =0.01), when BAV phenotypes were compared with the frequency of AS in phenotype 3 being higher than phenotype 1, whereas frequency of AI in phenotype 1 was found to be higher than other phenotypes. The frequency of the mass or vegetation in phenotype 1 was found to be significantly lower, in comparison to other phenotypes (*P*=0.001). Although, we found a higher frequency of co-arctation of aorta and mitral valve prolapse in phenotype 1 patients, there was no significant statistical difference between different BAV phenotypes, when these two disease entities were assessed.

**Table 3:** Valvular finding data by type of BAV phenotype

	**Phenotype1**	**Phenotype2**	**Phenotype3**	**Phenotype5**	**P-value**
Diastolic dysfunction	14(8.6%)	2 (8%)	9 (12.2%)	2 (5.7%)	0.701
AS	53 (32.5%)	6 (24%)	44 (59.5%)	19 (54.3%)	<0.001
Mild	11 (6.7%)	1 (4%)	4 (5.4%)	Zero	
Moderate	16 (9.8%)	1 (4%)	16 (21.6%)	6 (17,1%)	
Severe	26 (16%)	4 (16%)	24 (32.4%)	13 (37.1%)	
AI	135 (82.8%)	15 (60%)	52 (70.3%)	22 (62.9%)	0.007
Mild	26 (16%)	1 (4%)	8(10.8%)	6 (17.1%)	
Moderate	49 (30.1%)	5 (20%)	24 (34.2%)	4 (11.4%)	
Severe	60 (36.8%)	9 (36%)	20 (27%)	12 (34.3%)	
MR	14 (18.9%)	3 (12%)	4 (24.5%)	7 (20%)	0.460
Mild	17 (10.4%)	2 (8%)	8 (10.8%)	2 (5.7%)	
Moderate	16 (9.8%)	1 (4%)	Zero	2 (5.7%)	
Severe	7 (4.3%)	Zero	6 (8.1%)	3 (8.6%)	
TR	15 (9.2%)	5 (20%)	10 (13.5%)	Zero	0.052

AI: aortic insufficiency, AS: aortic stenosis, MR: mitral regurgitation, TR: tricuspid regurgitation

Further comparative analysis was done after combining the data set of phenotypes 1 and 2 representing BAV-AP (188 patients, 62.5%) and phenotypes 3, 4 and 5 representing BAV-RL (112 patients, 37.5%). The average age of BAV-AP and BAV-RL groups were 39± 15 and 40± 17 years respectively. There was no significant statistical difference between the two groups as far as age is concerned. In BAV-AP group, 72.9% of patients were male and in BAV-RL group, 70.5% of patients were male with no significant statistical difference between the two groups. It was evident that the BAV- AP patients had higher number of normal aorta and type 2 aortopathy (*P*<0.01). Tables 4 and 5 demonstrate echocardiographic and valvular findings for BAV-AP and BAV-RL. BAV-RL patients when compared to BAV-AP patients had higher frequencies of dilated aortic arch (25% vs. 4.3%, *P* <0.001), AS (56.3% vs. 31.4%, *P* <0.001), mass or vegetation on AV (14.3 vs. 6.4%, *P=*0.023) and lower frequencies of dilated aortic root (42.9% vs. 57.4%, *P=*0.01), AI (68.8% vs. 79.8%, *P=*0.034) and co-arctation of aorta (3.6% vs. 11.7%, *P=*0.01).

**Table 4:** Echocardiography findings in phenotypes BAV-AP and BAV-RL

	**BAV-RL**	**BAV-AP**	**P value**
LVEDD	5.24±1.00	5.38±1.06	0.22
Abnormal LV	44 (39.3)	85 (45.2)	0.31
RVEDD	3.28±0.32	3.21±0.3	0.06
Abnormal RV	33 (29.5)	43 (29.9)	0.2
Abnormal LA	31 (27.7)	35 (18.6)	0.06
Abnormal RA	6 (5.4)	2 (1.1)	0.02
IVSD	10.93 (2.22)	10.65±2.42	0.31
LVH	57 (50.9)	116 (61.7)	0.2
Mild	48 (42.9)	64 (34)	
Moderate	6 (5.4)	5 (2.7)	
Severe	1 (0.9)	3 (1.6)	
Annulus of aorta	2.46±0.33	2.44±0.37	0.67
Dilated annulus of aorta	15 (13.4)	21 (11.2)	0.56
Aortic root	3.45±0.40	3.57±0.49	0.03
Dilated aortic root	48 (42.9)	108 (57.4)	0.01
STJ size	3.21±0.50	3.17±0.48	0.51
Dilated STJ size	36 (32.1)	70 (37.2)	0.37
Ascending aorta	3.97±0.77	3.95±0.74	0.79
Dilated AAO	79 (70.5)	133 (70.7)	0.96
Aortic arch	2.80±0.42	2.58±0.35	<0.001
Dilated aortic arch	28 (25)	8 (4.3)	<0.001
LVEF	50.07‏±9.69	50.97±8.38	0.39
Low LVEF	22 (19.6)	34 (18.1)	0.73
TAPSE	21.28±2.67	21.71±2.27	0.13

Data are showns as mean (SD) or No. (%). AAO: ascending aorta, IVSD: inter-ventricular septum diameter, LA: left atrium, LV: left ventricle, LVEF: left ventricular ejection fraction, LVEDD: left ventricular end diastolic diameter, LVH: left ventricular hypertrophy, RA: right atrium, RV: right ventricle, RVEDD: right ventricular diameter, STJ: sinotubular junction, TAPSE: tricuspid annular plane systolic excursion.

**Table 5:** Valvular finding data by phenotypes BAV-AP and BAV-RL

	**BAV-AP**	BAV-RL	P-value
Diastolic dysfunction	16 (8.5%)	11 (9.8%)	0.743
G1	7 (3.7%)	8 (7.1%)	
G2	7 (3.7%)	2 (1.8%)	
G3	2 (1.1%)	1 (0.9%)	
AS	51 (31.4%)	63 (56.3%)	< 0.001
Mild	12 (6.4%)	4 (3.6%)	
Moderate	17 (9%)	22 (19.6%)	
Severe	30 (16%)	37 (33%)	
AI	150 (79.8%)	77 (68.8%)	0.034
Mild	27 (14.4%)	16 (14.3%)	
Moderate	54 (28.7%)	28 (25%)	
Severe	69 (36.7%)	33 (29.5%)	
MR	43 (22.9%)	24 (21.4%)	0.774
Mild	19 (10.1%)	10 (8.9%)	-
Moderate	17 (9%)	5 (4.5%)	
Severe	7 (3.7%)	9 (8%)	
TR	2 (10.6%)	11 (9.8%)	0.823
Mild	18 (9.6%)	6 (5.4%)	
Moderate	2 (1.1%)	4 (3.6%)	
Severe	Zero	1 (0.9%)	
CoA	22 (11.7%)	4 (3.6%)	0.010
Mass or vegetation on AV	12 (6.4%)	16 (14.3%)	0.023
MVP	13 (6.9%)	1 (0.9%)	0.014

AI: aortic insufficiency, AP: antero-posterior, AS: aortic stenosis, AV: aortic valve, BAV: bicuspid aortic valve, CoA: coarctation of aorta, MR: mitral regurgitation, MVP: mitral valve prolapse, RL: right-left, TR: tricuspid regurgitation

## Discussion

In this study, various phenotypes of BAV and associated aortopathies were identified by echocardiographic examination in 300 BAV patients who were known to our institution. Most of the patients were male (72%) and their age ranged from 14-76 years. As, previous studies in literature suggested, the ratio of male to female involvement in our cohort was 3:1.^1,2^ Similar incidence for male involvement in BAV was also reported by previous studies.^11,13,18^ In our study, when BAV-AP and BAV-RL were compared, we found no significant differences in prevalence of pathologies with respect to age and sex; The most common phenotypes were phenotype 1 and 3 and the prevalence of phenotype 4 was found to be a mere 1%, in agreement with Kang et al findings.^11^ In our study, BAV-AP and BAV-RL, constituted 62% and 37.3% of the BAV cases respectively. While, most studies confirm our findings with respect to frequency of different BAV phenotypes, in literature there is only one study, which did not report higher prevalence of BAV-AP phenotype.^19^ In Kang et al study, the prevalence of BAV-AP and BAV-RL was 55.7% and 44.3% respectively with no differences in age or in the frequency of male sex.^11^ Previous studies in literature have described a spectrum of non- valvular cardiovascular pathologies associated with BAV, namely; coarctation of the aorta, anomalies of the coronaries, aneurysm of sinus of Valsalva, aortic aneurysm, dissection of aorta, supra-valvular aortic stenosis (AS), patent ductus arteriosus (PDA), ventricular septal defect (VSD), Shone complex, familial aneurysm syndrome, dissection of thoracic aorta and Turner syndrome.^20^ In our study, PDA and VSD were observed only in one patient, coronary artery anomalies in 4 patients, aneurysm of sinus of Valsalva in 4 patients and coarctation of the aorta in 26 patients. Additionally, assessment of our data revealed that, patients with BAV-RL phenotype were more likely to have associated dilatation of aortic arch, AS and mass and vegetation on AV. Recent study results in literature, concerning potential relationship between BAV phenotypes and valvular dysfunction appear to be contradictory. Of note, valvular function does not appear to be impaired in all patients with the diagnosis of BAV and valvular dysfunction in patients with BAV seems to exhibit a wide clinical spectrum of patterns. Some patients have an isolated AS, while others present with single-cuspid aortic cusp prolapse, leading to aortic insufficiency (AI) in the absence of stenosis. Additionally, concurrent AS and AI in BAV patients is not uncommon.^13,21^ In our study it was evident that BAV-RL phenotype compared to BAV-AP, was significantly more likely to be associated with AS, but no difference in frequency of AI was observed when the two BAV phenotypes were compared. This is in agreement with Kang et al study, suggesting that the moderate to severe AS in BAV-RL (66.2% vs 46.2%), and moderate to severe AI in BAV-AP (32.3% vs 6.8%) were the predominant patterns of valvular dysfunction.^11^ It is widely known that BAV patients have an increased risk of aortic dilatation and dissection. However, the pathogenesis of the formation of aortic aneurysm in these patients is still unclear. The association between aortopathies and different phenotypes of BAV can be attributed to either genetic.^5,22^ or biomechanical factors caused by, increased asymmetrical shear stress on the aortic wall induced by, eccentric turbulent flow through the BAV. And this pathological flow can in turn explain why, different BAV phenotypes could lead to variable segmental dilatation patterns of aorta.^23-28^ For instance, in BAV-AP and BAV-RL, the abnormal blood flow pattern caused by differential orientation of the valve cusps is directed towards the right-anterior and right-posterior aortic walls respectively. In the study conducted by Mahadevia et al, this abnormal helical flow pattern was explained as the reason for; dilatation of the aortic root only (type 1) or the entire ascending aorta and arch (type 3) in the majority of BAV-RL patients. Patients with BAV-AP, were shown to exhibit dilatation of tubular of ascending aorta (type 2) in their study.^15^ In our study, we observed that, BAV-RL phenotype was associated with lower number of type 2 aortopathy when compared to BAV-AP. Further assessment of our data have shown that there was a clear difference between the two BAV phenotypes, when aortic arch diameter was studied. However, there was no noticeable difference in diameter of aortic root and ascending aorta between the two phenotypes. The Kang et al, study results suggested that, normal aorta was the most common phenotype in BAV-AP patients (33.3% vs. 18.9%) and type 3 aortopathy was the most common phenotype in BAV-RL patients (40.5% vs. 9.7%). Kang also reported no difference in dimensions of ascending aorta when BAV-AP and BAV-RL are compared.^11^ In the study, conducted by Mahadevia et al, type 1 and 3 aortopathy were present in most of BAV-RL cases, and absent in most patients with BAV-AP.^15^ In a retrospective study, conducted by Schaefer et al, the relationship between BAV phenotypes, aortic dimensions and the elastic properties of aorta were studied. Schaefer et al found that patients with BAV-AP phenotypes had a larger diameter, at sinuses of Valsalva and smaller diameter at the arch when compared to BAV-RL phenotypes. Also, the higher frequency of AI in BAV-AP patients in Schaefer study was attributed to larger and stiffer sinuses of Valsalva in this group of patients.^18^ On the other hand the study by Buchner and colleagues produced conflicting results with no significant difference in aortic diameter, when different BAV phenotypes were compared.^12^ A recent retrospective study without longitudinal follow up, conducted by Habchi et al, has also produced conflicting results with the dimension of the ascending aorta at presentation was not different between the BAV phenotypes after adjustment for age, gender, body surface area (BSA), and the presence of moderate or severe aortic valve disease. Nevertheless, BAV-RL patients were marginally more likely to have an aortic root aneurysm. (86% vs. 78%, *P*=0.043). Habchi et al study results suggested a strong univariate associations between older age, male gender, taller height, heavier weight, greater body mass index and BSA and increased aortic dimensions, and this was found to be independent of BAV phenotype. They also, concluded that, patients with dilated aortic root, are more likely to be male, have lower BSA, with lower frequency of moderate to severe AS, and increased frequency of moderate AI.^29^

Another interesting study by Kinoshita et al, evaluated the risk factors for aortic dilatation in Japanese patients with BAV, who had previously undergone aortic valve replacement (AVR), especially focusing on potential impact of valve phenotypes. The results of this single center retrospective study suggested that the aortic growth rate of ascending aorta was similar between the BAV phenotypes. But the larger size of the ascending aorta (>40 mm) and presence of more than moderate AI at the time of the AVR, were found to be strong predictors of future aortic dilatation.^30^

Since it is only prudent to follow up the more susceptible group of patients more diligently, the ultimate aim of clinical studies, assessing the frequency and types of aortopathy in BAV phenotypes, is mainly the recognition of the patients at risk for catastrophic aortic rupture. Smaller sample size, limited inclusion criteria resulting in selection bias, lack of standardization of classifications used for BAV phenotypes and possible errors in the measurements of aortic dimensions are the primary limitations of current studies found in the literature. The use of Z scores described by Campens (normalized aortic dimensions adjusted for age, gender and body surface) and analysis of the rate of expansion of aortic diameter (mm/year) will further standardize the data-set acquired by centers involved in BAV investigations.^31^ Essentially, for evidence-based management of patients with diagnosed BAV; multicenter well characterized BAV cohort with long-term follow-up and a registry based on standardized classification of BAV phenotypes are needed. Additionally, risk stratification based on family and medical history of BAV patients, inheritance patterns of BAV patients, and most importantly information derived from imaging data, is crucial, for appropriate development of management strategies for this group of patients.

In addition to proper identification of patients at risk of aortic rupture, it is of pivotal importance to determine the most useful diagnostic modality for longitudinal follow-up of BAV patients. Transthoracic echocardiography is the imaging technique of choice for initial diagnosis of BAV patients. For accurate classification of BAV phenotypes, determination of cusp separation and the site of cusp fusion, transesophageal echocardiography or other imaging modalities may also be required. Recently 3-D echocardiography has demonstrated greater sensitivity and specificity for diagnosis of BAV phenotypes when compared to 2-D echocardiography ^32^

Additionally, especially in patients with heavy calcifications, both multi-detector computed tomography (MDCT)^11^ and cardiovascular magnetic resonance imaging (CMR) have been proven to be of great utility in assessment of anatomy of aortic valve and pathologies of ascending aorta.^12,33^

## Conclusion

According to the results of our study, there is a significant association between BAV phenotypes and different patterns of valvular disorders and aortopathies. These findings suggest that examining leaflet morphology in BAV might help in risk stratification of these patients.

## Ethical approval

The study was approved by the institutional review board of Tabriz University of Medical Sciences.

## Competing interests

All authors declare no competing financial interests exist.

## Acknowledgments

We sincerely thank Kathirvel Subramaniam for his help in editing the manuscript.
